# Validation of dosimetric field matching accuracy from proton therapy using a robotic patient positioning system

**DOI:** 10.1120/jacmp.v11i2.3015

**Published:** 2010-04-12

**Authors:** Jonathan B. Farr, Avril O'Ryan‐Blair, Frederick Jesseph, Wen‐Chien Hsi, Chris E. Allgower, Anthony E. Mascia, Allan F. Thornton, Andreas N. Schreuder

**Affiliations:** ^1^ Midwest Proton Radiotherapy Institute 2425 Milo B. Sampson Lane Bloomington Indiana USA; ^2^ Present address: Procure Treatment Centers, Inc. 2420 North Walnut Street Bloomington Indiana USA

**Keywords:** proton, matching, robotic, positioning

## Abstract

Large area, shallow fields are well suited to proton therapy. However, due to beam production limitations, such volumes typically require multiple matched fields. This is problematic due to the relatively narrow beam penumbra at shallow depths compared to electron and photon beams. Therefore, highly accurate dose planning and delivery is required. As the dose delivery includes shifting the patient for matched fields, accuracy at the 1–2 millimeter level in patient positioning is also required. This study investigates the dosimetric accuracy of such proton field matching by an innovative robotic patient positioner system (RPPS). The dosimetric comparisons were made between treatment planning system calculations, radiographic film and ionization chamber measurements. The results indicated good agreement amongst the methods and suggest that proton field matching by a RPPS is accurate and efficient.

PACS number: 87.55.km

## I. INTRODUCTION

It is possible with proton therapy to use the protons' range in media property to spare underlying structures for large length/area shallow target volumes. This specific application usually requires field matching. Proton beams are excellent in this regard because of their relatively narrow penumbra at shallow depths compared to other tele‐therapy beam types. Conversely, it is the relatively narrow penumbra that puts additional importance on the accuracy of the field match borders.

This study investigates the accuracy of proton field matching by a robotic patient positioner system (RPPS) for proton beams in dosimetric terms. In general, any radiotherapy patient positioning system capable of high accuracy can perform adequate field matching. This investigation uses a RPPS and intends to demonstrate the validity of that approach. Because of the high dose gradients involved with proton therapy at shallow to moderate depths, multiple match junctions between the matched fields are sometimes used to reduce the resulting small volumes of relatively high dose (“hot spots”). In practice, the multiple match junctions are changed in a specific order through the entire treatment course. This practice is sometimes termed “feathering.” This investigation also includes dosimetric validation of this technique using rectilinear physics fields.

## II. MATERIALS AND METHODS

### B.1 Facility

The Midwest Proton Radiotherapy Institute (MPRI) currently treats patients with a nominal 208 MeV proton beam in three treatment rooms developed by the Indiana University Cyclotron Facility. Two of the treatment rooms include isocentric gantries with a new uniform beam scanning dose delivery system.^(1)^ The other treatment room uses a traditional Fixed Horizontal Beam Line (FHBL) with a double passive scattering system and fixed range modulator.^(2)^ ^,^ ^(3)^ ^,^ ^(4)^ That beamline is similar to other passive systems currently in clinical use.^(5)^ ^,^ ^(6)^ This work was performed entirely in the FHBL room. However, assuming that the target is stationary, the principles are the same for uniform scanning systems.

### B.2 Robotic patient positioner

The FHBL room takes advantage of a novel RPPS that was built using an industrial robot (Motoman, Inc., West Carrollton, Ohio: model UP 200).^(7)^ The robot has a specified accuracy of ± 150 microns when transiting up to a 200 kg payload. This accuracy, combined with six degrees of freedom, provides the RPPS excellent potential for accurate and precise field matching.

### B.3 Study plannings

Comparison was made to calculated dose distributions from a pencil beam algorithm performed with a commercial treatment planning system (TPS) (Computerized Medical Systems, Inc., St. Louis, Missouri: model FOCUS Radiation Treatment Planning System with Proton Planning Capability).

The FHBL is limited to a maximum field size of about 19 cm diameter. However, at the time of this work the maximum field size was 10 cm diameter. This restriction resulted in relatively more matched fields delivered to patients. Although this restriction has been eased, multiple matching is still used at the facility.

This study has two components. First, the accuracy of field matching dosimetry and the possible benefit of feathering are explored as they are generally used. Secondly, the evaluation method is applied to a complicated patient case that required four matched fields. As discussed later, feathering was not considered to be clinically required in this case. It does serve, however, as a good evaluation of complicated multifield matching with the RPPS.

#### Matched physics fields

**Figure 1:**
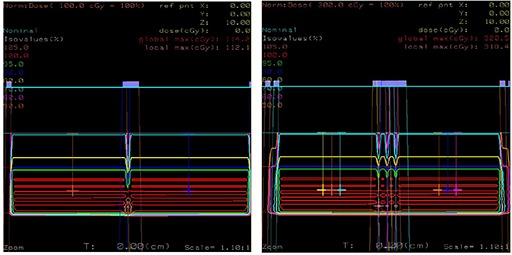
Proton treatment plan for laterally matched rectilinear fields F1A and F2A5: (Left) the fields are abutted radiologically at 7 cm depth; (Right) the fields F1A and F2A5 are weighted 1/3 of their total for feathering −7.5 mm, 0 mm, and +7.5 mm.

The first case for the field matching study consisted of two rectilinear proton fields (F1A and F2A5) defined by physical apertures. The defining aperture openings were 10 cm in length and 4 cm in width with the inside edge between the fields designed to be matched (Fig. 1, Left). A 7 cm range in water was used with a 4 cm spread‐out Bragg peak (SOBP). All fields were matched at 5 cm depth in water, corresponding to the center of the SOPB. The second case considered was to use the fields F1A and F2A5 and feather them by 0 and ±7.5 mm to determine the effect on the maximum dose in the match junction volume (Fig. 1, Right).

#### Matched Patient Fields

**Figure 2:**
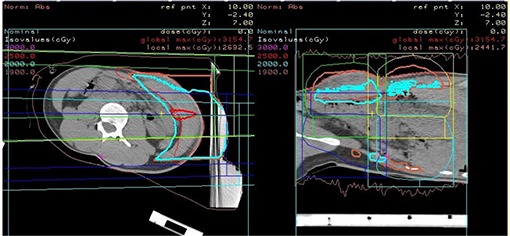
Proton treatment plan showing 100% dose delivery (cyan) in the axial plane. An effect of the ranged proton beam was to pull the dose delivery off the underlying parenchyma. The range compensator was required to tailor the distal end of the dose distribution to the target volume as well as accounting for the curved patient abdomen. Proton treatment plan showing 100% dose delivery (cyan) in the sagittal plane.

**Figure 3:**
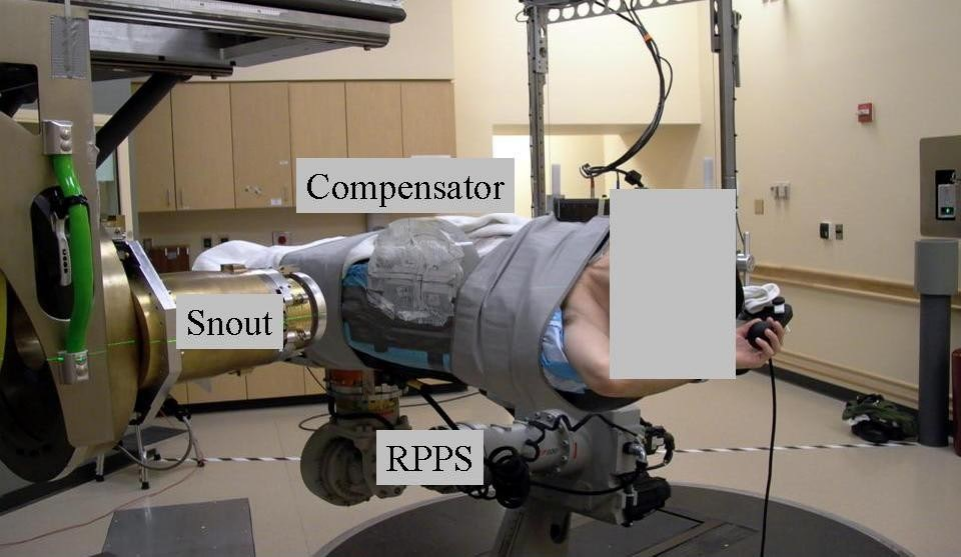
Photograph of the clinical setup for a four‐field RPPS match delivery.

The applied clinical case was from a patient treatment course. The patient required a relatively large, shallow clinical volume to be treated, approximately 18 cm by 14 cm at the surface and extending to a depth of 4 cm. In this case, to provide full dose coverage, the fields were matched in the bolus above the patient. Due to the initial limited field size, constraints of the FHBL four matched proton fields at extended source to surface distance (SSD) were required to achieve target volume coverage. Figure 2 presents the planning for the patient case and Fig. 3 illustrates the treatment setup in the FHBL room. In the latter figure (from left to right), is the proton beam snout with custom aperture setup for extended SSD and air gap. The oversize range compensator was included as part of the patient's immobilization with the patient and immobilization positioned by the RPPS. The range compensator form was estimated from a preliminary plan and then built into the immobilization. A subsequent CT planning scan was obtained of the patient, immobilization and fixed range compensator, and imported back into the TPS for verification.

### B.4 Dosimetry

Two types of ionization chambers were used, a commercial A150 parallel plate chamber (PTW, Freiburg, Germany; model: Markus A150), and a small 0.7 cm^3^ thimble chamber (NAC‐mini) fabricated in house from Vespel from a design developed at the National Accelerator Center, South Africa.^(8)^ The Markus chamber was used for longitudinal measurements and delivery system dose calibration. The mini‐chamber was used for field edge characterization. The ion chamber scans were collected using a water dosimetry phantom (Wellhöfer, Schwarzenbruck, Germany: model WP700).

Radiographic film was used as an integrating dosimeter for relative dosimetry characterization (Kodak, Rochester, New York; type: X‐Omat V). The film exposures were performed within blocks of polystyrene at the water equivalent thickness, and developed and scanned with a commercial film scanner (Vidar Systems Corporation, Herndon, Virginia; model VXR‐16).

### B.5 Calibration

The Markus A150 received calibration from an accredited dose calibration laboratory (University of Wisconsin Accredited Dosimetry Calibration Laboratory, Madison, Wisconsin). The NAC‐mini holds an in‐house relative calibration to the calibrated Markus chamber.

(1) $$SD=(OD-{OD}_{base+fog})/({OD}_{maximum}-{OD}_{base+fog})$$

The film calibration was based on a series of exposures of varying dose over the range 1–180 cGy. In this study, the concept of standard density (SD) (Eq. 1) was used where SD is the related to the optical density (OD), and where OD is the base 10 logarithm of optical transmittance. The OD calculation accounts for the limits of maximum OD and minimum OD corrected for base and fog.^(9)^ The calibration was performed using a series of seven calibration exposures made with a 3 cm physical diameter aperture. The relatively low lateral scatter and weak dependence of output factor on field size for protons with respect to photons or electrons permitted this technique. The primary fields delivered to films for the physics and patient fields were also correlated to ionization chamber measurements in the WP700 under equivalent conditions.

#### Matched Physics Fields

The two rectilinear fields F1A and F2V5 were delivered sequentially to the film. Between irradiations, the RPPS was used to move the film in the polystyrene stack to the matched position. The equivalent polystyrene thickness was chosen as being representative of the field matching depth in water.

#### Matched Patient Fields

The four treatment fields were delivered to the film in polystyrene. The RPPS was used to move the film that had been placed on the treatment couch in a stack of polystyrene of 9 cm water equivalent depth (including the bolus equivalent thickness). This depth was chosen as being representative of the water equivalent maximum dose depth from the TPS for the study. For the patient case investigation, an additional method was used for comparison. Use of the NAC‐mini permitted relatively high resolution scans to be acquired. A two‐dimensional scan grid was performed at 1 mm and 10 mm spacing in the cross‐plane and in‐plane directions, respectively. Due to the complexity required to scan the four individual fields as a whole, the fields were scanned individually and numerically patched together by the RPPS offset vectors used for the treatment delivery. This simplification is thought to be acceptable based on the submillimeter positioning accuracy of the RPPS. In total, a matrix of 270 by 32 scans comprised of 8640 individual data points are used to form the ionization scan data set.

## III. RESULTS & DISCUSSION

### C.1 Calibration

#### Spot exposure calibration

**Figure 4:**
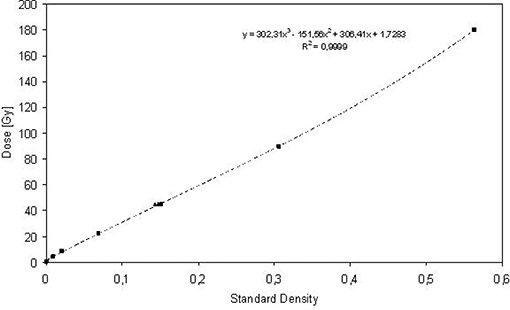
Calibration of film density to proton dose as determined by ionization chamber dosimetry; □ are the individual film dose calibrations; Δ are the central field deliveries.

**Figure 5:**
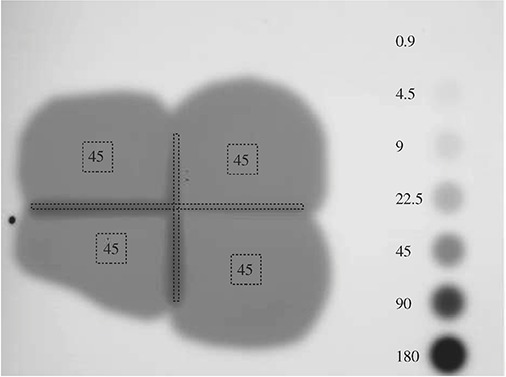
Proton exposures of treatment fields (4) and calibration fields (7). The regions of interest used for the analysis were the calibration fields, the centers of the four treatment fields indicated by the black square outlines, and the elongated rectangular outlines representative of the field matches.

The results of the calibration are presented in Tables 1 and 2, and Figs. 4 and 5. The relationship between dose and SD is nonlinear at low doses, as expected from the multiple hit model.^(9)^ ^,^ ^(10)^ ^,^ ^(11)^ The data fit well using a third order polynomial function, giving accuracy of 2% or better for doses above 22.5–180.0 cGy. For the field matching study, lower doses were non relevant.

**Table 1:** Results of film dose calibration: Calibration spot exposures.

*Dose [cGy]*	*Transmissivity*	*Standard Density*	*Dose Calculation [cGy]*	*Dose Calc./Dose*
180.0	2.105	0.563	180.0	1.000
90.0	1.533	0.305	89.8	0.998
45.0	1.268	0.151	45.6	1.013
22.5	1.146	0.069	22.2	0.987
9.0	1.080	0.020	7.9	0.878
4.5	1.065	0.009	4.5	1.000
0.9	1.053	0.000	1.9	2.111

**Table 2:** Results of film dose calibration: Calibration treatment field exposures.

*Dose [cGy]*	*Transmissivity*	*Standard Density*	*Dose Calculation [cGy]*	*Dose Calc./Dose*
45.0	1.256	0.143	43.4	0.964
45.0	1.264	0.149	45.0	1.000
45.0	1.262	0.147	44.5	0.989
45.0	1.264	0.149	44.9	0.998

#### Primary patient field comparison

For the multiple patient fields, the polynomial function was then used to back‐calculate dose from SD for the calibration deliveries of known doses. This result is presented in Table 1. The accuracy for this calculation was less than or equal to 2% for doses above 22 cGy. The doses calculated from SD measurements at the centers of each treatment field (Table 2) also show good agreement with the ionization chamber dosimetry. The repeatability (precision) within 4% for the four calculated treatment field doses is taken to be representative of the repeatability of the technique at a particular SD.

### C.2 Matched physics fields

**Figure 6:**
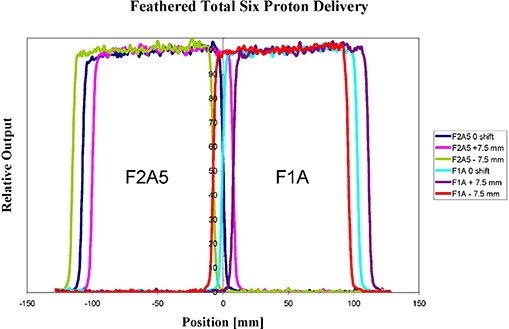
Normalized ionization scans at 6.3 cm depth in water of two matched rectilinear fields feathered −7.5, 0, +7.5 mm with 1/3 dose weighting.

**Figure 7:**
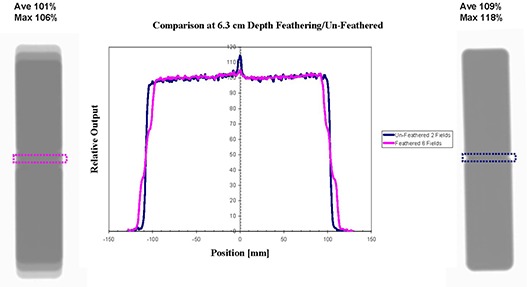
Normalized ionization scans of feathered and nonfeathered matched rectilinear fields (center), and radiographic film dosimetry results for feathered (left) and nonfeathered (right) deliveries of the same physical fields. Water and equivalent depth is 6.3 cm.

It is illustrative to observe how, in practice, the feathering technique functions. Figure 6 presents composite results from ionization scans at 6.3 cm water depth of the fields F1A and F2V5 feathered 0 and ±7.5 mm mm with 1/3 weighting. The feathering distance was chosen to be about two penumbra widths at this depth. The depth of 6.3 cm was chosen for investigation because it corresponds to dose maximum calculated by the TPS. As observed, the field edge penumbra is spoiled by the feathering but the interior edge dose is also reduced for the hot spot reduction. The TPS indicates the maximum dose for fields F1A and F2V5 to be 114% when they are weighted 100% each without feathering. With feathering, the maximum calculated dose reduces to 106%. Ionization scan results at a depth of 6.3 cm are presented in Fig. 7. The water phantom ionization chamber scans are summed and renormalized to illustrate the effect at the match junction from feathering. The data indicate the feathering effect has reduced the relative dose in the hot junction volume from an average of 115% to 102%. Figure 7 also shows the film exposure results for the un‐feathered fields (right) and feathered fields (left). The SD conversion to dose indicates a reduction from 109% average and 118% maximum in the junction to 101% and 106%, respectively. The maximum values are for a single pixel.

#### C.3 Matched Patient Fields

Figure 5 depicts the film scan with regions of interest (ROI) indicated and doses in cGy from ionization chamber dosimetry reported, where known. Using this approach the field matching dose in the ROI's was evaluated. The average volume isodose level in those regions at 9 cm depth was determined to be 135% when normalized to the center field treatment level, and the maximum was 160%.

**Figure 8:**
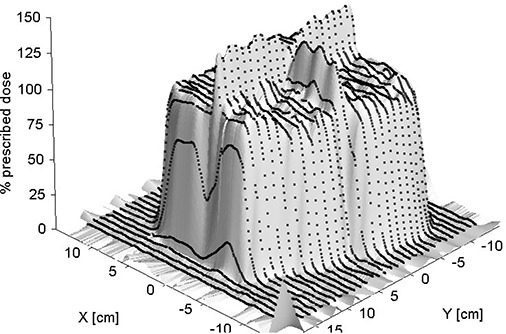
Visualization of 2D data at depth of 9 cm in water phantom

A data visualization of the ionization chamber data is presented as Fig. 8. Maximum and average chamber dose measurements are presented in Table 3. The Table also serves to compare calculations from the treatment planning system and film dosimetry results. The average relative dose results within the four‐field matching volumes compare within 4% with a maximum of 128% between the three methods. In this case, the comparision was thought to be clinically acceptable as the volume lay entirely within the target. Further reduction of the hot spot could have been performed by using a RPPS kick to align the proton field edges. Clinically it was decided not to due this because of the additional complication to the treatment therapists (i.e. only orthogonal shifts were used). The maximum relative doses were not thought to be clinically relevant as they are representative of small volumes on the order of a few pixels or voxels. The trend of these maxima can be understood, however, by recalling that the film and treatment planning minimum dimensions for consideration are comparable and relatively small with regard to the ionization chamber volume.

**Table 3:** Results of dose calculation and measurement intercomparison.

*Type*	*Av. Rel. Dose*	*Max. Rel. Dose*
Planning Calculation	130%	160%
Film Dosimetry	126%	149%
Ionization Dosimetry	128%	142%

## V. CONCLUSIONS

Proton therapy is suitable for treating volumes of relatively large area and shallow depth. In this way the ranged property of the protons can spare underlying structures. However, proton field matching may be required. This investigation considered matching proton fields with a high precision RPPS. The precision of the RPPS facilitates daily field offset (feathering) that distributes setup and beam alignment uncertainty among the treatment fractions, in addition to reducing the relative hot volume junction dose at depth. Validation by ionization chamber scans in water and film dosimetry indicates agreement with the TPS within the accuracy of the methods (2–5%). In addition, a complicated four‐field clinical field matching case was considered using dose calculation, 2D ionization chamber scans, and film dosimetry. The results between the three methods agree within 7% and indicate an average of 135% dose in the junction area. In summary, the dosimetric results from field matching by a RPPS were clinically acceptable.

## ACKNOWLEDGEMENTS

This work was conducted in a facility constructed with support from Research Facilities Improvement Program Grant C06 RR17407‐01 from the National Center for Research Resources, National Institutes of Health. The authors would also like to thank Miles Wagner of the Francis Burr Proton Therapy Center for fabricating the large custom compensator used for the four‐field patient treatment.
